# Prescribing workload administration to optimise isothermic heat acclimation

**DOI:** 10.1186/2046-7648-4-S1-A83

**Published:** 2015-09-14

**Authors:** Oliver R Gibson, Peter W Watt, Neil S Maxwell

**Affiliations:** 1 Centre for Sport and Exercise Science and Medicine (SESAME), University of Brighton, Eastbourne, UK

## Introduction

Repeated exercise-heat exposures, known as heat acclimation (HA), are often implemented as an intervention to attenuate decrements in physiological strain and exercise tolerance prior to work in normothermic and hot, humid conditions. The fundamental potentiating stimuli for thermoregulatory adaptation are repeated, significant rises in core temperature. Targeting of a specific core temperature is known as isothermic, or controlled hyperthermic HA. Different methods of modulating the exercise component of isothermic HA have been implemented, with prescription previously based upon either peak oxygen uptake (VO_2peak_), power, or subjective ratings of perceived exertion or thermal sensation. Interestingly, metabolic heat production, a measure to determine changes in core temperature, has not been used to prescribe isothermic HA. The aim of this study was to determine the relationship between the rate of rectal (core) temperature (Trec) increase, and different methods for prescribing workload during an acute exercise-heat exposure, with the objective of trying to refine the prescription of isothermic HA workloads.

## Methods

Following preliminary testing to determine VO_2peak_, fifty four male participants (Age 23.1(4.2) years, Height 180(6) cm, Mass 76.3 (10.1) kg, Body surface area (BSA) 1.95 (0.13) m^2^, Body Fat 13.8 (4.1) %, VO_2peak _3.82 (0.66) L.min^-1^) cycled in uncompensable heat stress 40 °C, 39 %rh for 29 (2) min to replicate the active stage of a typical isothermic HA session. The initial exercise workload was prescribed based upon a %VO_2peak _(59.7 ± 8.7). Potential exercise prescription variables corresponding to the actual %VO_2peak _performed were retrospectively calculated following each session as follows mean power (W.kg^-1^), power (%max), metabolic heat production (MHP; W.kg^-1^), MHP/BSA (W.m^-2^), heat production (H_prod_; W.kg^-1^), H_prod_/BSA (W.m^-2^), VO_2 _(%peak), heart rate (HR; %max), rating of perceived exertion (RPE) and thermal sensation (TS). Pearson product moment correlations were calculated for each prescriptive workload variable against the rate of change in Trec (°C.hr^-1^) occurring in each session. Linear regression was then performed to determine the workload prescription required to increase Trec by 1.5°C within 30 minutes thus replicating an increase from resting (37.0°C) to the isothermic target (38.5°C) temperature.

## Results

All variables demonstrated significant (*p <*0.05) relationships with the rate of change in Trec in ranked order as follows: power (r^2 ^= 0.58; 2.1(0.1) W.kg^-1^), MHP (r^2 ^= 0.50; 10.8(2.7) W.kg^-1^), MHP/BSA (r^2 ^= 0.46; 419(97) W.m^-2^), power (r^2 ^= 0.46; 51 (12) %max), H_prod _(r^2 ^= 0.43; 8.7 (2.1) W.kg^-1^), H_prod_/BSA (r^2 ^= 0.39; 336(77) W.m^-2^), RPE (r^2 ^= 0.33; 14(2)), HR (r^2 ^= 0.28; 83(10) %max), VO_2 _(r^2 ^= 0.28; 50.8(10.5) %peak), and TS (r^2 ^= 0.10; 6.3(0.6)). Linear regression described the workload prescription to increase Trec by 1.5°C in 30 minutes as follows power = 2.7 W.kg^-1^, MHP = = 12.9 W.kg^-1^, MHP/BSA = 518 W.m^-2^, power = 64 %max, H_prod _= 10.9 W.kg^-1^, H_prod_/BSA = 422 W.m^-2^, RPE = 17, HR = 95 %max, VO_2 _= 68 %peak, and TS = 8.

## Discussion

Rapid (<30 min) attainment of the target Trec for isothermic HA optimises the efficiency of this intervention. Prescription of isothermic HA intensity utilising power (W.kg^-1 ^or %max) or H_prod _(W.kg^-1 ^or W.m^-2^) have the strongest relationship with the rate of change in Trec. This is likely due to the direct relationship with the heat balance equation. The calculated workload prescriptions can therefore, be implemented by practitioners to accurately increase Trec.

## Conclusion

Prescribing HA workloads utilising power (W.kg^-1 ^or %max), MHP (W.kg^-1 ^or W.m^-2^), or H_prod _(W.kg^-1 ^or W.m^-2^) demonstrates least variability of the methods tested. Utilising relative power (W.kg^-1^) or MHP and H_prod _to prescribe isothermic HA removes the requirement for a pre HA VO_2peak _test.

